# Influence of Microstructure and Chemical Composition on Microhardness and Wear Properties of Laser Borided Monel 400

**DOI:** 10.3390/ma13245757

**Published:** 2020-12-16

**Authors:** Mateusz Kukliński, Aneta Bartkowska, Damian Przestacki, Grzegorz Kinal

**Affiliations:** 1 Faculty of Mechanical Engineering, Institute of Mechanical Technology, Poznan University of Technology, ul. Piotrowo 3, 61-138 Poznan, Poland; mateusz.kuklinski@doctorate.put.poznan.pl; 2 Faculty of Materials Engineering and Technical Physics, Institute of Materials Science and Engineering, Poznan University of Technology, ul. Jana Pawła II 24, 61-138 Poznan, Poland; aneta.bartkowska@put.poznan.pl; 3 Faculty of Civil and Transport Engineering, Institute of Machines and Motor Vehicles, Poznan University of Technology, Piotrowo 3, 61-138 Poznan, Poland; grzegorz.kinal@put.poznan.pl

**Keywords:** Monel 400, laser heat treatment, laser boriding, diode laser, wear resistance, wear mechanism

## Abstract

In this study, wear properties of Monel 400 after laser alloying with boron are described. Surfaces were prepared by covering them with boron paste layers of two different thicknesses (100 µm and 200 μm) and re-melting using diode laser. Laser beam power density was equal to 178.3 kW/cm^2^. Two laser beam scanning velocities were chosen for the process: 5 m/min and 50 m/min. Surfaces alloyed with boron were investigated in terms of wear resistance, and the surface of untreated Monel 400 was examined for comparison. Wear tests were performed using counterspecimen made from steel 100Cr6 and water as a lubricant. Both quantitative and qualitative analysis of surfaces after wear test are described in this paper. Additionally, microstructures and properties of obtained laser alloyed surfaces are presented. It was found that the wear resistance increased from four to tens of times, depending on parameters of the laser boriding process. The wear mechanism was mainly adhesive for surfaces alloyed with initial boron layer 100 µm thick and evolves to abrasive with increasing boron content and laser beam scanning velocity. Iron particles detached from counterspecimens were detected on each borided surface after the wear test, and it was found that the harder the surface the less built-ups are present. Moreover, adhered iron particles oxidized during the wear test.

## 1. Introduction

Nowadays, non-ferrous alloys have an increasingly important role in industry and their properties are often modified with high-energy heat sources like laser beam [1,2,3]. Laser processes, including laser alloying [4,5,6] and laser remelting [3] are increasingly displacing plasma processes [7] due to the fact that quality and properties of obtained layers are promising. Monel 400, which was laser treated in this study, is a one-phase nickel-copper alloy. Its main advantage is high corrosion resistance. Thus, this alloy is generally applied in harsh environments, including seawater, alkalis, salts or acids. The main industries in which parts made from Monel 400 are present (as valves, pumps and tanks) are marine engineering and chemical processing [8,9,10]. On the other hand, the main disadvantage of Monel 400 is its relatively low hardness in comparison with other nickel alloys [11]. Taking into consideration that products made from this material are often exposed to high flow rates, there is a risk of failures due to erosion, cavitation or wear.

Wear of materials generates high production costs and thus it is often a research object in materials engineering [12]. Due to a high risk of wearing parts made from Monel, tribological behavior of this alloy has been the subject of research for at least 50 years. In 1973, Bill reported that wear mechanism of Monel 400 alloy is initially adhesive with plastic deformation and the next stage of wear is an oxidation process occurring on the surface [13]. Nowadays researchers focus on investigating wear of Monel 400 in different states and environments. In 2016, Esgin et al. examined wear properties of different Monel alloys produced with powder metallurgy. It was found that mass loss during the wear test under the same conditions was the highest for Monel 400 alloy. Moreover, only this alloy suffered adhesive wear during the process [14]. In 2018, Ma et al., investigated tribocorossion behavior of Monel 400 in artificial seawater with different loads applied during the wear test. Regardless of the load, small regions of loose corrosion were found on surfaces after the wear test and it was found that the wear was the dominant factor in the corrosive wear process. Moreover, abrasive wear occurred in each experiment [15]. In 2019, Waliszyn and Adamkiewicz investigated Monel 400 erosive wear under the influence of hydraulic cavitation. They found that (in these conditions) proneness to erosive damage of this Ni-Cu alloy is between pure nickel and steel C45 which means relatively poor wear resistance [16].

Low wear resistance of Monel 400 means improvements should be made to the tribological properties of its surface. One of the processes which increases hardness and thus wear resistance of nickel alloys is boriding. Researchers have been describing effects of alloying nickel and its alloys with boron for about two decades. In 2000, Ueda et al. borided surface of pure nickel with powder-pack method. The process resulted in increasing surface hardness about six times and significantly improved wear resistance [17]. After this successful attempt, researchers continued studies, working on new substrate materials and methods for improving tribological properties by boron alloying. For this purpose, Anthymidis et al. borided pure nickel in a fluidized bed reactor. As a result, produced coatings showed an approximately 50% increase in resistance to wear compared to the untreated nickel [18]. In further studies, it was found that boriding is an effective method of improving surface properties of nickel alloys as well. In 2008, Petrova et al. investigated effects of boriding alloys for automotive applications including Inconel 625 and Inconel 718. Microhardness increased six and four times respectively after the process [19]. In this study the comparison of wear resistance between treated and untreated surfaces was not considered, however, five years later, Sista et al. borided Inconel 600 electrochemically, resulting in 8 to 10 times increase in microhardness and examined the wear resistance. It was found that the wear volume dropped more than 40 times due to alloying surface with boron [20]. In 2017, Gunen et al. confirmed these results on Inconel 625 by obtaining improved wear resistance by boriding [21] and borotitanizing [22]. In 2019, Silva et al. reported similar results for Inconel 718 [23] and Gheisari et al. proved that boriding increases wear resistance of nickel alloys in elevated temperatures as well [24]. Diffusion boriding of Monel 400 surface was performed in 2019 and as a result of this process both hardness and wear resistance were improved by several times [25].

In this study boriding of Monel 400 was performed with laser boriding process. The main advantages of this method are: obtaining layers of greater thickness than diffusion boriding and a possibility to treat only selected areas instead of whole products. In 1996 Nakata et al. had already reported that laser boriding of Ni-Cu alloys (of different proportions of these elements) is an effective method for increasing hardness of their surfaces, and wear resistance was improved up to about 40 times in comparison with the substrate [4]. In 2016, Monel 400 was laser alloyed with nickel, chrome, silicon and boron. This combination of alloying elements led to obtain surface 7 times harder [5]. In 2018 it was found that laser boriding of Monel 400 significantly increases its hardness, but this increase is highly dependent on laser remelting parameters: mainly laser beam power, laser beam scanning velocity and amount of boron in the molten pool [6]. Despite there being no research on wear resistance of laser borided Monel 400, it is worth noting that researchers report improving other metal alloys’ wear resistance by alloying surfaces during this process [26,27,28,29,30]. Thus, due to the fact that Monel 400 is highly exposed to wear, the authors decided to investigate this effect on this specific Ni-Cu alloy as well.

## 2. Materials and Methods

### 2.1. Materials

In this experiment, Monel 400 was laser alloyed with boron. Monel 400 is a single-phase nickel-copper alloy and its chemical composition is given in Table 1. Dimensions of specimens prepared for laser treatment were 30 mm × 20 mm × 12 mm. Material was laser alloyed with boron layers 100 µm and 200 µm thick. Amorphous boron powder was deposited on surfaces of specimens as a paste, after mixing it with sodium water glass. Counterspecimens for wear tests were made from bearing steel 100Cr6. Chemical composition of 100Cr6 steel is given in Table 2. Its hardness is equal to 64 HRC, which is approximately 700 HV.

### 2.2. Laser Heat Treatment

**Figure 1:**
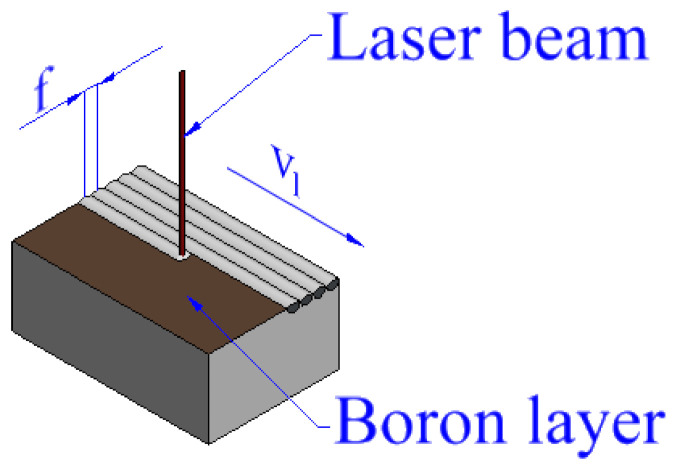
Scheme of the laser boriding process.

Laser heat treatment was performed using diode laser TRUMPF TruDiode 3006 which reaches 3 kW of power (TRUMPF, Ditzingen, Germany). Laser head was integrated with robot arm KUKA KR16-2 (KUKA, Augsburg, Germany) to manipulate location of the beam. To provide constant laser beam velocity on whole length of laser track, the laser beam was started above and turned off behind the specimen just like in papers [6,31]. The scheme of the laser alloying process is presented in Figure 1.

During laser alloying of Monel 400 with boron each laser track was produced parallelly to longer side of specimen and distance between tracks (f) was equal to 0.5 mm. Constant laser beam power P = 1400 W was used and considering the laser beam diameter was d_l_ = 1 mm, laser beam power density (q) was equal to 178.3 kW/cm^2^. Two laser scanning velocities were set: 5 m/min and 50 m/min. Laser heat treatment was performed on two different types of specimens: Monel 400 with initial boron layer of thickness equal to t_b_ = 100 µm and Monel 400 with boron layer of thickness equal to t_b_ = 200 µm. These parameters are collected in Table 3. The specimen marked with symbol “E” stands for untreated Monel 400.

### 2.3. Samples Preparation

After laser alloying, samples were cut for obtaining specimens for the wear test (with dimensions 13 mm × 10 mm × 6 mm). For comparison of the wear resistance, one additional specimen made from untreated Monel 400 was also prepared. Other pieces of borided samples were cut across laser tracks for obtaining metallographic microsections. Cutting was performed using hand saw blade to reduce any structural changes. Samples designed for wear test were slightly grinded to obtain Ra lower than 0.2 µm. Cross-sections of specimens designed for structural investigation were grinded with abrasive papers of grits ranged from 120 to 2000. Thereafter, they were polished for 20 min and etched with Marble’s reagent.

### 2.4. Microstructure and Chemical Composition

Microstructural investigation of laser borided layers’ cross-sections, as well as surfaces after wear tests was carried out using scanning electron microscope Tescan MIRA3 (TESCAN, Brno, Czech Republic). This instrument was equipped with Oxford Instruments EDS (Energy-dispersive X-ray spectroscopy) detector (Oxford Instruments, High Wycombe, UK) with AZtec system version 4.2, which were used for chemical composition analysis.

### 2.5. Microhardness Testing

Microhardness indentations were fabricated with Zwick 3212B Vickers tester (Zwick, Ulm, Germany) with constant load equal to 0.9807 N. Load was kept on samples for 15 s for each indentation. On each sample, indentations were made in different distances from surfaces, and five indentations were fabricated in the substrate.

### 2.6. Wear Test

**Figure 2:**
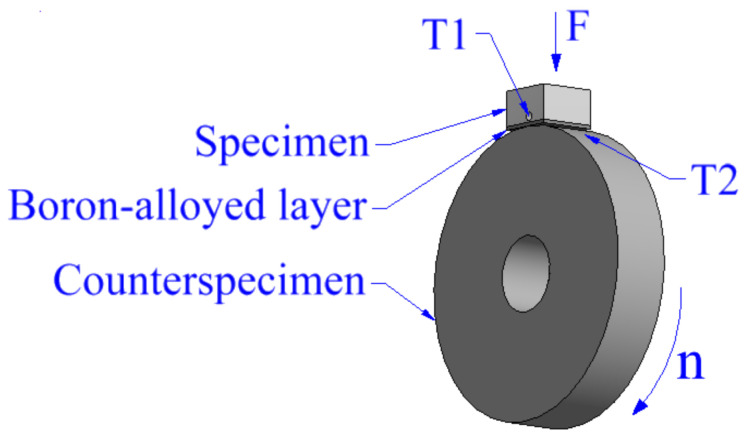
Scheme of the wear test.

Specimens cut to dimensions suitable for the wear tester were mounted in it in a way presented in Figure 2. Machine used for the testing was AMSLER A135 (Amsler, Szafuza, Switzerland) which allows to set specific load to the specimen and specific rotational speed to the counterspecimen. Dimensions of the counterspecimen were 45 mm in diameter and thickness equal to 12 mm, with mounting hole of diameter equal to 15 mm. In this study load was equal to F = 392 N and rotational speed of the counterspecimen n = 180 rev./min, just like at paper [32]. Some droplets of water were delivered to the friction zone to make the friction coefficient lower. During the process, two temperatures (T1 and T2) were measured using thermometer TES-1312A (TES, Taipei, Taiwan) with probe equipped with thermocouple type “K”—NiCr-NiAl. Places of these measurements are presented in Figure 2. Measurements of temperature T1 were taken from inside the sample through a drilled hole and temperature T2 was measured on surfaces of counterspecimens, just behind the friction zone. Moreover, frictional moments were measured for each specimen during the process. Testing time of boron-alloyed specimens was equal to t = 45 min. The wear test of sample made from Monel 400 was stopped after 5 min due to the fact that the specimen suffered notable seizing and large increases in temperatures T1 and T2 was observed.

### 2.7. Depth of Friction Zone and Mass Loss

For measuring depth of friction zone, contact profilometer ZEISS (Carl Zeiss, Oberkochen, Germany) was used. It was equipped with induction converter and SUFORM software version 2.0 provided by SAJD Metrologia company (SAJD, Kielce, Poland). Measuring the mass loss of specimens after the wear test was performed with laboratory weight Sartorius BP221S (PCE-instruments, Southampton, Hampshire, UK) with a precision of 0.0001 g.

## 3. Results and Discussion

### 3.1. Microstructural Characterization and Microhardness

**Figure 3:**
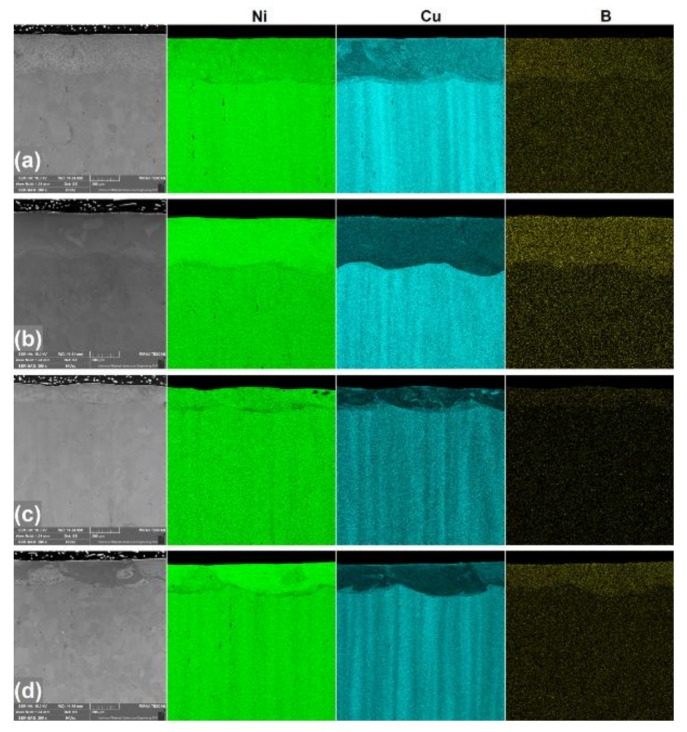
Laser borided layers produced on Monel 400 with different parameters with their EDS mappings: (**a**) v_l_ = 5 m/min and t_b_ = 100 µm, (**b**) v_l_ = 5 m/min and t_b_ = 200 µm, (**c**) v_l_ = 50 m/min and t_b_ = 100 µm, (**d**) v_l_ = 50 m/min and t_b_ = 200 µm.

**Figure 4:**
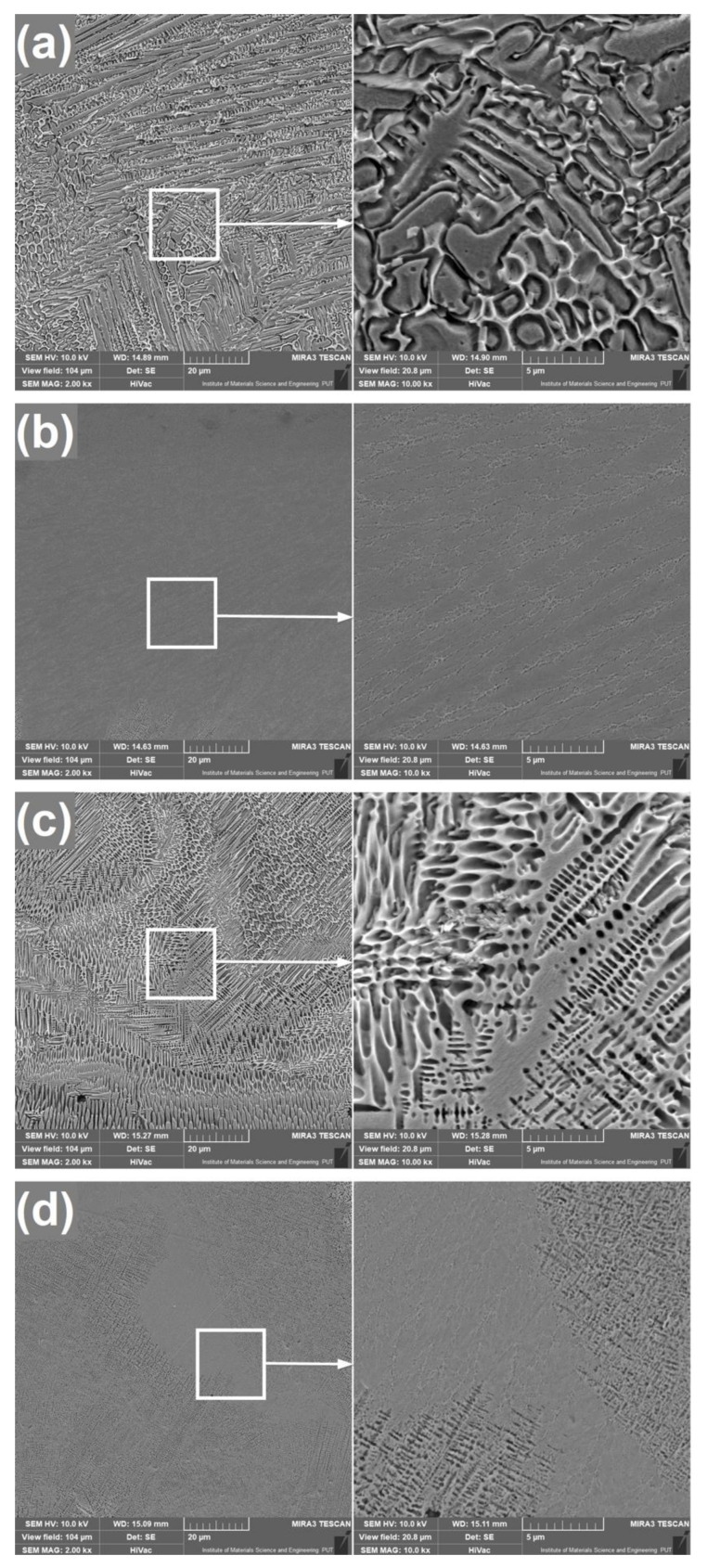
Microstructures of laser borided layers produced on Monel 400 with different parameters: (**a**) v_l_ = 5 m/min and t_b_ = 100 µm, (**b**) v_l_ = 5 m/min and t_b_ = 200 µm, (**c**) v_l_ = 50 m/min and t_b_ = 100 µm, (**d**) v_l_ = 50 m/min and t_b_ = 200 µm.

Microstructures of laser alloyed surfaces are shown in Figure 3 and Figure 4. Additionally, Figure 3 includes EDS mappings of produced coatings for chemical composition analysis. Each of these figures presents surfaces treated with two different laser beam scanning velocities and two different thicknesses of initial boron layer. Letters which are given within figures indicate following process parameters: (a) laser beam scanning velocity v_l_ = 5 m/min and initial boron layer thickness t_b_ = 100 µm, (b) v_l_ = 5 m/min and t_b_ = 200 µm, (c) v_l_ = 50 m/min and t_b_ = 100 µm, (d) v_l_ = 50 m/min and t_b_ = 200 µm.

It is clearly seen that laser re-melting of Monel 400 with boron has a significant influence on its microstructure. However, microstructures of layers with different initial boron thicknesses differ more from each other than layers produced with different laser beam scanning velocities. Layers alloyed with boron paste 100 µm thick, which are shown in Figure 3a,c and Figure 4a,c are built mainly of column crystals solidified in much directions which depend on turbulences of liquid metal during re-melting. Grains can be seen as lengthy or as equiaxed due to the fact that some of their crystallization fronts were directed parallel to the laser movement and it can be especially seen in Figure 4a. According to Safonov et al. [27], laser alloyed layers which include low amount of boron crystalize into structures of primary dendrites and a boride eutectic between them. It is considered that layer produced with v_l_ = 5 m/min is built of hypoeutectic and layer produced with v_l_ = 50 m/min is eutectic, because of higher concentration of boron in the re-melted volume. Moreover, it can be seen with high magnification in Figure 4a,c that sizes of grains are strongly dependent on laser beam scanning velocity. Column crystals emerged on specimen treated with v_l_ = 5 m/min are visibly few times larger than these obtained during alloying with v_l_ = 50 m/min. This effect is the result of grain growth during long time of exposition to laser radiation on specimens produced with v_l_ = 5 m/min and quick solidification of layers produced with v_l_ = 50 m/min. On the other hand, microstructures of surfaces which were alloyed with initial boron layer thickness equal to 200 µm are more uniform. Safonov et al. [27] concluded that microstructures laser alloyed with high amount of boron consist of primary boride crystals and a eutectic. In this study, these layers, in most of their volume, seem like solid one-phase materials which is a hypereutectic structure. However, in both cases there are noticeable areas of various tints in which chemical compositions and crystal orientations differ from other volume. Their presence is the result of insufficient mixing of substrates in the molten pool. This effect can be observed in EDS mappings presented in Figure 3. Although boron is distributed uniformly within produced coatings, concentrations of nickel and copper vary in different areas. Moreover, since the mixing is weaker in layers produced with higher laser beam scanning velocity (Figure 3c,d), these areas differ more from surrounding volume than in specimens produced with v_l_ = 5 m/min. This suggests that, due to insufficient mixing in coatings produced with v_l_ = 50 m/min, the microstructure is built of both hypereutectic and eutectic areas. Furthermore, it is worth noting that in both layers produced with 200 µm initial boron layer structure built of columnar crystals is visible between re-melted zone and the substrate.

Microstructures of layers alloyed with boron using different laser beam scanning velocities do not differ as significantly as if boron content is considered. However, the main difference between them is their depth. In this experiment depths of boron-alloyed layers were measured ten times for each specimen. Average depths of coatings produced using laser beam scanning velocity v_l_ = 5 m/min are equal to approximately 300 µm and 410 µm if initial boron layer thickness was 100 µm and 200 µm respectively. Increasing laser beam scanning velocity to 50 m/min results in obtaining layers enriched with boron approximately 130 µm and 175 µm deep. Both laser beam scanning velocity and initial boron layer thickness affect the depth of re-melting. The higher laser beam scanning velocity is, the shallower alloyed layer is. It is the result of shorter time of influence of laser radiation on material’s surface during the process. On the other hand, the more boron is provided in the molten pool, the deeper the layer that is obtained. This effect is considered to be caused by higher thermal conductivity of nickel borides than of Monel 400.

**Figure 5:**
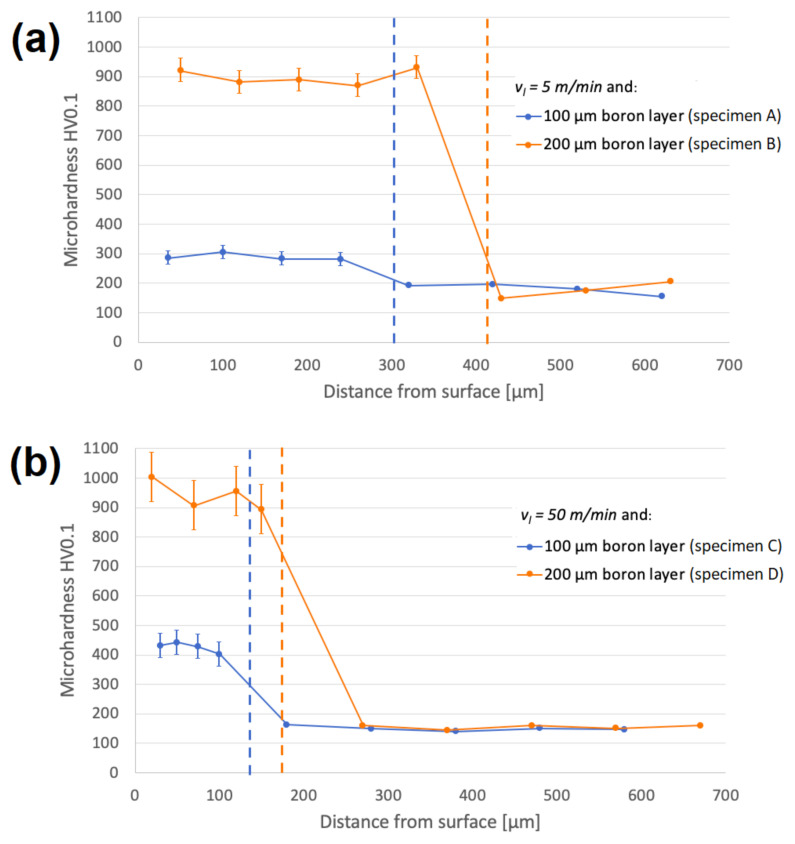
Microhardness of borided layers in different distances from surface, alloyed with: (**a**) v_l_ = 5 m/min and (**b**) v_l_ = 50 m/min.

Microhardness of untreated Monel 400 is ranged between 150 and 200 HV0.1. It was confirmed that alloying Monel 400 with boron increases these values significantly. In this study, as in case of depth, final results depend on laser beam scanning velocity and thickness of initial boron layer. In this study microhardness was measured in two different laser tracks. Average values of microhardness, depending on distance from surface, are presented in Figure 5. Laser alloying with initial boron layer 100 µm thick using laser beam scanning velocity equal to 5 m/min resulted in obtaining almost two times harder surface layer (between 280 and 310 HV0.1). Increasing boron content to initial layer thickness equal to 200 µm leads to obtain layer of microhardness between 870 and 930 HV01. Due to the fact that increasing laser beam scanning velocity results in decreasing depths of obtained layers, microhardness of surfaces alloyed with boron using v_l_ = 50 m/min is higher with the same initial boron content. The reason for this is higher concentration of hard nickel borides in smaller re-melted volume of material. Thus, microhardness of layer enriched with 100 µm of boron paste ranged between 400 and 450 HV0.1 if the laser beam scanning velocity is equal to 50 m/min. Doubling the initial boron content to 200 µm results in obtaining microhardness from 890 to 1000 HV0.1. Taking into consideration that surfaces treated with higher laser beam scanning velocity are built of smaller grains, their higher microhardness increase could also be affected by the higher number of grain boundaries present in re-melted zones.

### 3.2. Wear Analysis

**Figure 6:**
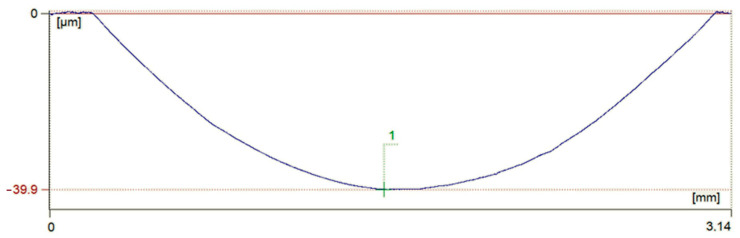
Measurement of depth of friction zone on specimen laser borided with v_l_ = 50 m/min and t_b_ = 100 µm.

**Figure 7:**
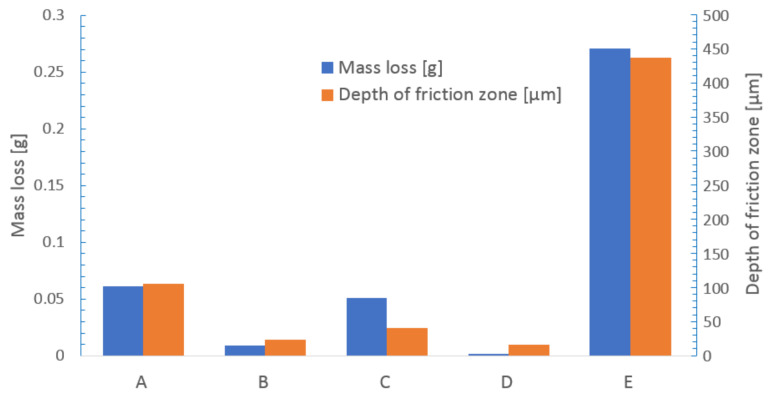
Mass losses and depths of friction zones of specimens after the wear test.

For quantitative comparison of wear resistance of laser borided Monel 400 surfaces, two indicators were taken into consideration: depth of the friction zone and mass loss of each specimen after the wear test. In Figure 6, an exemplary measurement of depth of friction zone is shown. This specific profile was measured on sample borided with 100 µm boron paste using laser beam scanning velocity equal to 50 m/min. Figure 7 is a graphic representation of values of friction zones’ depths and mass losses after the wear test.

It was found that the rate of wear is strongly dependent on microhardness. The shallowest friction zone after the wear test was measured on sample laser alloyed with 200 µm boron paste using laser beam scanning velocity equal to v_l_ = 50 m/min. In this case, the depth of friction zone was equal to 15.8 µm. The second lowest value, equal to 23.7 µm, was measured on sample which was prepared with the same boron content but with lower laser beam scanning velocity v_l_ = 5 m/min. Samples produced with initial boron layers 100 µm thick suffered wear which resulted in obtaining friction zones 39.9 µm and 105.5 µm deep on samples alloyed using laser beam scanning velocity equal to 50 m/min and 5 m/min respectively. For comparison, depth of friction zone on untreated Monel 400 was equal to 436.5 µm after only 5 min of wear test. These numbers confirm that laser alloying of Monel 400 surface with boron significantly increases its resistance to wear. In terms of differences in depth of the friction zone, the resistance to wear was increased minimum 4-times and maximum 27-times, and even more, if the fact that sample of untreated Monel 400 was tested for 9-times shorter period of time is taken into consideration.

Values of mass loss after the wear test are convergent with these described above. The lowest mass loss, measured on specimen laser alloyed with 200 µm boron paste and v_l_ = 50 m/min, was equal to 0.0013 g after 45 min of testing. Mass loss of sample re-melted with same boron content but slower laser beam scanning velocity v_l_ = 5 m/min was equal to 0.009 g. Mass loss, as well as depth of friction zone, was greater if initial boron layer thickness was equal to 100 µm. In these cases, masses of specimens decreased by 0.0511 g if laser beam scanning velocity v_l_ = 50 m/min and 0.0611 g if v_l_ = 5 m/min was used for the boron-alloying process. Mass loss of untreated Monel 400 was equal to 0.2707 g after 5 min of testing. These values indicate even greater increase in wear resistance than values of friction zones’ depths. The lowest measured value of mass loss, on specimen tested for 45 min, is more than 200-times lower than mass reduction of Monel 400 sample after 5 min of the wear test. On the other hand, quadrupled increase in wear resistance of surface alloyed with 100 µm of boron paste and laser beam scanning velocity equal to 5 m/min, in terms of mass loss, is comparable with the result obtained by analysis of depths of friction zones. Nonetheless, these results clearly indicate that laser alloying Monel 400 with boron significantly enhances wear resistance of the material’s surface.

**Figure 8:**
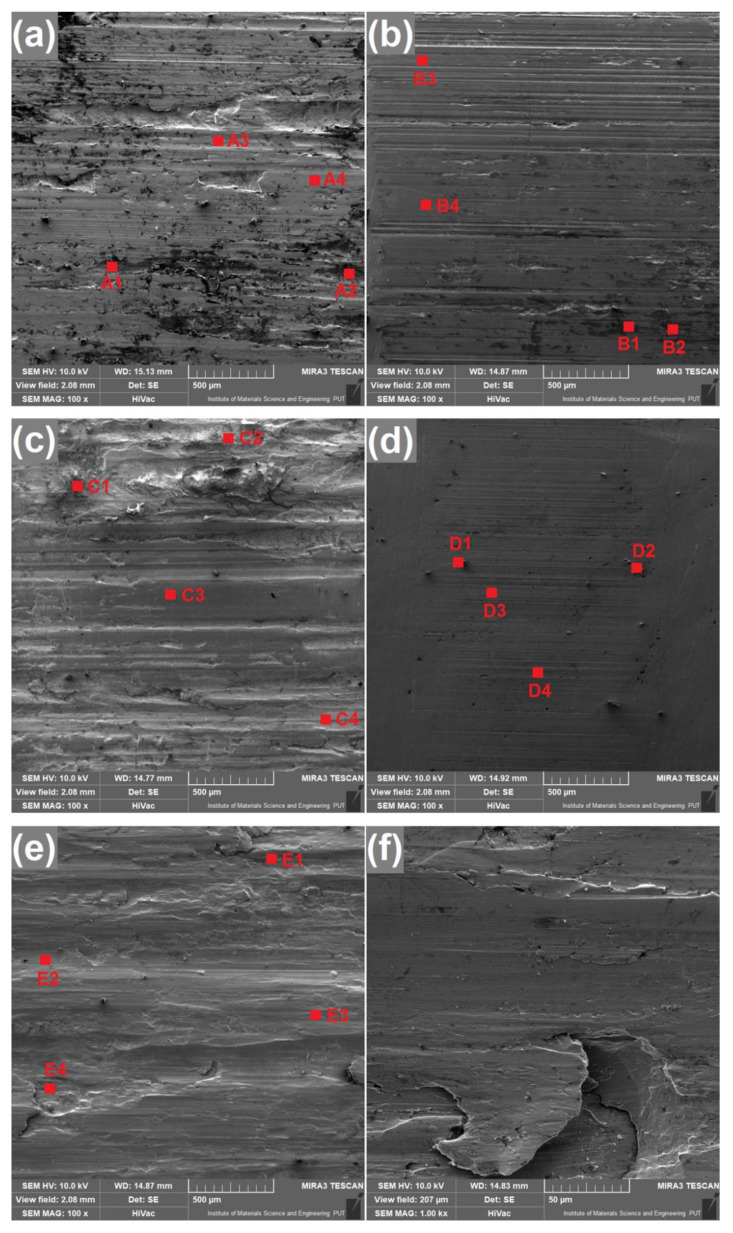
Surfaces after the wear test of specimens produced with: (**a**) v_l_ = 5 m/min and t_b_ = 100 µm, (**b**) v_l_ = 5 m/min and t_b_ = 200 µm, (**c**) v_l_ = 50 m/min and t_b_ = 100 µm, (**d**) v_l_ = 50 m/min and t_b_ = 200 µm, (**e**),(**f**) untreated Monel 400.

**Figure 9:**
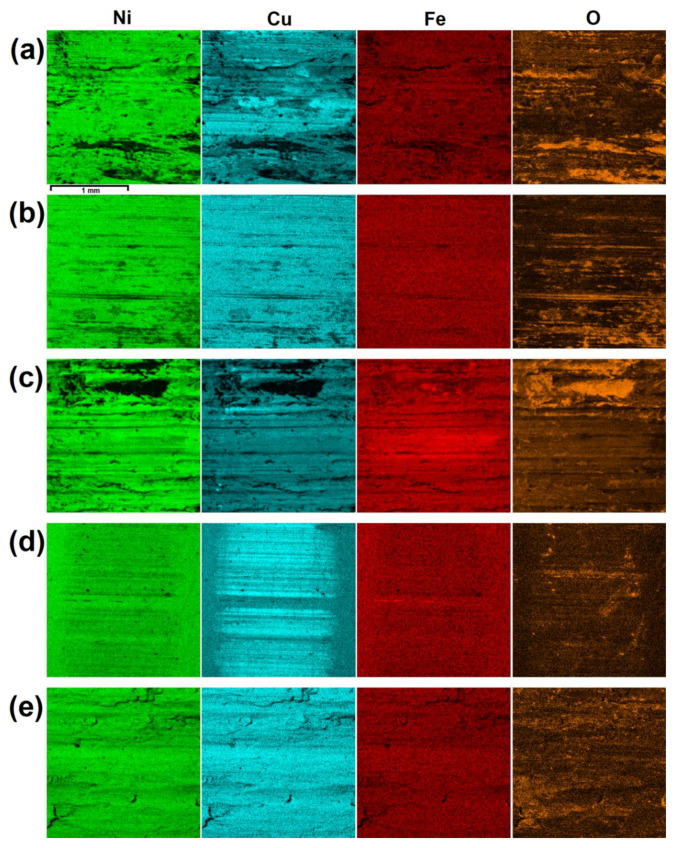
EDS mappings of surfaces after the wear test: (**a**) v_l_ = 5 m/min and t_b_ = 100 µm, (**b**) v_l_ = 5 m/min and t_b_ = 200 µm, (**c**) v_l_ = 50 m/min and t_b_ = 100 µm, (**d**) v_l_ = 50 m/min and t_b_ = 200 µm, (**e**) untreated Monel 400.

**Figure 10:**
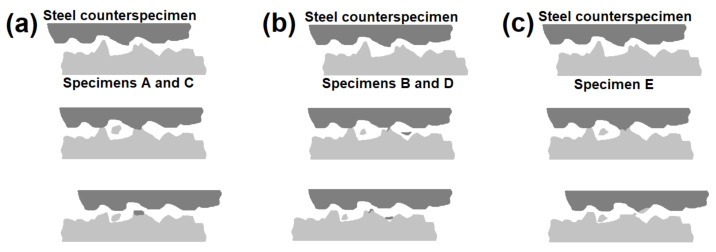
Schemes of wear mechanisms on different specimens: (**a**) Specimens A and C: serious abrasive wear of the specimen and adhesive wear of the counterspecimen, (**b**) Specimens B and D: minor abrasive wear of the specimen and adhesive wear of the counterspecimen, (**c**) Specimen E: serious abrasive and adhesive wear of the specimen, with plastic deformation.

For qualitative assessment of surfaces after the wear test, microscopic examination using scanning electron microscope was performed as well as chemical composition analysis. Results of these observations are given in Figure 8 and Figure 9. In each figure sliding direction is vertical. EDS mappings were taken from the same areas as corresponding photos in Figure 8. Red dots present in Figure 8 represent spots analyzed in terms of chemical composition and their results are given in Table 4. Letters given in Figure 8 and Figure 9 indicate specimens as in Table 3. Analysis of chemical composition, both for points and mappings, was limited to four elements: nickel, copper, iron and oxygen, because verification of their presence is sufficient for the assessment of the nature of wear. Results shown in bold font in Table 4 represent spots of increased content of iron or oxygen. Moreover, for better understanding of wear mechanisms which occurred between specimens and counterspecimens, a schematic view of phenomena in wear zones is shown in Figure 10.

After the wear test, surfaces laser alloyed with boron paste 100 µm thick (Figure 8a,c) differ from these which were laser alloyed with higher boron content. Surface presented in Figure 8a which was produced using laser beam scanning velocity equal to 5 m/min contains much irregularities after the wear test. Moreover, in pits and built-ups visible on the surface, an increased amount of iron and oxygen was spotted and results of this analysis are given in Table 4 as symbols A1 and A2. The confirmation of this effect is shown in Figure 9a—areas which are darker in Figure 8a contains less nickel and copper but more oxygen than surrounding zones. Taking into consideration chemical composition of Monel 400 alloy and 100Cr6 steel, it is considered that detected fragments is iron detached from counterspecimen, which reacted with oxygen during the wear test. Presence of grooves and iron particles on surface presented in Figure 8a suggests that this surface suffered serious abrasive wear. Surface which was alloyed with the same boron content but higher laser beam scanning velocity that is presented in Figure 8c appears similar after the wear test. However, in this case there are fewer built-ups including iron and oxygen which are visible in Figure 9c and in spots with symbols C1 and C2. On the other hand, more wide scratches were found. This suggests that higher hardness resulted in reducing tendency to adhere iron particles from counterspecimen and wear mechanism became more abrasive than adhesive. Schematic representation of wear mechanism of specimens alloyed with 100 µm- thick boron paste is shown in Figure 10a.

**Figure 11:**
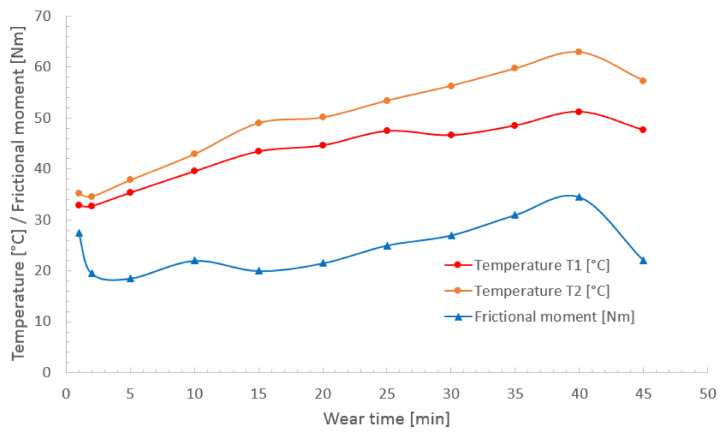
Frictional moment and temperatures T1 and T2 as function of wear time for specimen laser borided with laser beam scanning velocity v_l_ = 50 m/min and initial boron layer t_b_ = 100 µm.

It is clearly seen in Figure 8b,d that specimens which were alloyed with boron paste 200 µm thick suffered minor wear in comparison with these described above. Both surfaces contain much fewer grooves and signs of plastic deformation. Surface presented in Figure 8b is mostly covered with scratches of various widths but there are considerably fewer deformed areas than on surfaces alloyed with 100 µm boron paste. Despite this, iron particles were detected on this surface as well but in a smaller amount which can be seen in Figure 8b and in results from spots marked as B1 and B2. These results lead to the conclusion that in this case the major mechanism of wear was abrasive. However, presence of iron built-ups detected during analysis of chemical composition suggests that slight adhesive wear also occurred in some areas. Moreover, an exemplary graph of frictional moment and temperatures during the wear test of specimen laser alloyed with 200 µm boron paste and laser beam scanning velocity equal to 5 m/min (Figure 11) indicates irregular movement resistance. On the other hand, a specimen produced with the same amount of boron but higher laser beam scanning velocity which surface is presented in Figure 8d. This suffered only insignificant wear in comparison with other samples. Scratches visible after the wear test are shallow and narrow, and there are no signs of plastic deformation of alloyed layer. Foreign particles are visible but only as spots (not surfaces like on other specimens) and in small amount. These spots of increased oxygen concentration are visible in Figure 9d, and results of their chemical composition analysis are shown in Table 4 as D1 and D2. This suggests that laser alloyed layer with the highest microhardness suffered only minor abrasive wear, with insignificant share of adhesive wear and oxidation. The scheme of this wear mechanism is shown in Figure 10b.

Finally, it is worth noting that although surface of untreated Monel 400 suffered the most serious plastic deformation during the wear test, areas with increased iron concentration were not detected on its surface. It is proven with results shown in Figure 9e and spots marked as E1–E4 in Table 4. In this case, only minor oxidation distributed uniformly was detected. The reason of these effects is lower hardness of Monel 400 itself than hardness of the steel counterspecimen. It is considered that in this case material was detached from the Monel 400 specimen. An example of area of plastic deformation which could occur as a result of detachment of material from Monel 400 surface is given in Figure 8f with higher magnification. The condition of Monel 400 surface after the wear test ongoing for only 5 min, in comparison with other specimens which were tested for 9 times longer period of time, is a notable evidence that laser alloying with boron is an effective method for increasing wear properties of this nickel-copper alloy. This wear mechanism is shown schematically in Figure 10c.

## 4. Conclusions

On the basis of results and examination of condition of laser borided Monel 400 after wear tests following conclusions were formulated:

(1) Laser alloying with boron is an effective method for improving wear resistance of Monel 400 and level of this enhancement is strongly dependent on microhardness of obtained layers and hence on the laser boriding parameters. This fact confirms that laser boriding can be implemented to increase hardness and wear properties of this specific Ni-Cu alloy, as it is for other nickel-based metals.
(2) Wear resistance, both in terms of depth of friction zone and mass loss after the wear test, improves with increasing initial boron content and laser beam scanning velocity during the laser boriding process. In this study, the highest wear resistance was observed for surface alloyed with initial boron layer 200 µm thick using laser beam scanning velocity equal to 50 m/min.
(3) The improvement of wear resistance achieved by laser alloying Monel 400 with boron is quantitatively larger than increase of microhardness in each examined case. For example, microhardness of surface laser borided with 100 µm thick initial boron layer using laser beam scanning velocity equal to 50 m/min almost doubles while mass loss during the wear test decreases about five times in comparison with untreated Monel 400.
(4) In conditions chosen for this examination, mechanism of wear evolves from severe abrasive and adhesive on surface alloyed with initial boron layer 100 µm thick using laser beam scanning velocity 5 m/min to insignificant abrasive wear of surface re-melted with 200 µm boron layer and laser beam scanning velocity equal to 50 m/min. Thus, increasing boron content and laser beam scanning velocity leads to reduction of adhesive wear on alloyed surfaces.

## Figures and Tables

**Table 1:** Chemical composition of Monel 400.

Cu [%]	Si [%]	Fe [%]	Mn [%]	C [%]	S [%]	Ni
31	0.5	2.5	2.0	0.3	0.024	bal.

**Table 2:** Chemical composition of 100Cr6 bearing steel.

C [%]	Si [%]	Mn [%]	Cr [%]	Mo [%]	Cu [%]	Al [%]	P [%]	S [%]	Fe
1.00	0.25	0.35	1.45	0.1	0.3	0.05	0.025	0.015	bal.

**Table 3:** Laser alloying parameters.

P [W]	q [kW/cm^2^]	t_b_ [µm]	v_l_ [m/min]	d_l_ [mm]	f [mm]	Symbol of Specimen
1400	178.3	100	5	1	0.5	A
1400	178.3	200	5	1	0.5	B
1400	178.3	100	50	1	0.5	C
1400	178.3	200	50	1	0.5	D
-	-	-	-	-	-	E

**Table 4:** Results of chemical composition analysis of spots marked in Figure 8.

**Symbol**	**A1**	**A2**	**A3**	**A4**	**B1**	**B2**	**B3**	**B4**	**C1**	**C2**
Ni [Wt%]	**30.4**	**26.9**	59.7	42.9	**37.1**	**38.1**	57.0	58.2	**19.3**	**19.9**
Cu [Wt%]	**18.2**	**16.9**	35.3	53.4	**7.6**	**7.3**	33.9	33.0	**16.4**	**0.8**
Fe [Wt%]	**20.1**	**21.6**	3.1	2.2	**28.7**	**26.9**	6.4	6.5	**25.6**	**44.7**
O [Wt%]	**31.3**	**34.7**	1.8	1.5	**26.6**	**27.7**	2.3	2.2	**38.8**	**34.6**
**Symbol**	**C3**	**C4**	**D1**	**D2**	**D3**	**D4**	**E1**	**E2**	**E3**	**E4**
Ni [Wt%]	58.3	59.9	**49.4**	**41.3**	60.5	62.7	61.1	63.2	64.1	63.5
Cu [Wt%]	30.0	32.2	**23.3**	**45.0**	36.0	34.0	33.1	33.7	30.7	31.5
Fe [Wt%]	8.4	3.7	**4.6**	**1.8**	2.1	2.3	2.7	2.4	4.0	2.2
O [Wt%]	3.4	4.1	**22.6**	**11.9**	1.4	1.0	3.1	0.7	1.3	2.8
